# Dietary Proteins as Determinants of Metabolic and Physiologic Functions of the Gastrointestinal Tract 

**DOI:** 10.3390/nu3050574

**Published:** 2011-05-11

**Authors:** Alireza Jahan-Mihan, Bohdan L. Luhovyy, Dalia El Khoury, G. Harvey Anderson

**Affiliations:** Department of Nutritional Sciences, Faculty of Medicine, University of Toronto, Toronto, ON, M5S 3E2, Canada; Email: alireza.jahanmihan@utoronto.ca (A.J.-M.); bohdan.luhovyy@utoronto.ca (B.L.L.); dalia.elkhoury@utoronto.ca (D.E.K.)

**Keywords:** protein, gastrointestinal tract, metabolism, physiology

## Abstract

Dietary proteins elicit a wide range of nutritional and biological functions. Beyond their nutritional role as the source of amino acids for protein synthesis, they are instrumental in the regulation of food intake, glucose and lipid metabolism, blood pressure, bone metabolism and immune function. The interaction of dietary proteins and their products of digestion with the regulatory functions of the gastrointestinal (GI) tract plays a dominant role in determining the physiological properties of proteins. The site of interaction is widespread, from the oral cavity to the colon. The characteristics of proteins that influence their interaction with the GI tract in a source-dependent manner include their physico-chemical properties, their amino acid composition and sequence, their bioactive peptides, their digestion kinetics and also the non-protein bioactive components conjugated with them. Within the GI tract, these products affect several regulatory functions by interacting with receptors releasing hormones, affecting stomach emptying and GI transport and absorption, transmitting neural signals to the brain, and modifying the microflora. This review discusses the interaction of dietary proteins during digestion and absorption with the physiological and metabolic functions of the GI tract, and illustrates the importance of this interaction in the regulation of amino acid, glucose, lipid metabolism, and food intake.

## 1. Introduction

Ingested proteins have a wide range of nutritional and biological functions. Nutritionally, they are essential sources of amino acids and can provide energy. Traditionally, their function and quality have been judged primarily on their ability to provide essential amino acids and support protein synthesis. However, this focus fails to recognize their influence on many regulatory systems including those affecting amino acid, glucose and lipid metabolism, bone metabolism, blood pressure, immune function, food intake and body weight. Physico-chemical properties, amino acid composition and bioactive peptides encrypted in amino acid sequences of proteins influence mechanical, hormonal and neuroendocrine functions of the gastrointestinal (GI) tract. Therefore, the purpose of this review is to discuss the qualities of dietary proteins that affect their actions during ingestion, digestion and absorption on the physiological and metabolic functions of the GI tract.

In the following, the characteristics of dietary proteins that influence physiology and metabolism are reviewed first, followed by an examination of their fate in the GI tract and how they determine the role of the GI tract in regulating metabolism and food intake. The final discussion proposes that traditional methods of addressing protein quality and amino acid requirements need to take into account the functionality of proteins beyond their role in protein synthesis. 

## 2. Chemical and Structural Characteristics of Dietary Proteins

The physiological consequences of ingesting dietary proteins are determined by their food source, composition, and matrix and processing.

### 2.1. Food Source

The primary sources of dietary proteins are muscle, milk, egg and plant proteins, and within each source is a complex mixture of proteins that affect physiologic regulation in different ways when consumed as a mixture or as purified proteins.

*Muscle proteins* originate from meat products including red meat, fish and poultry and are divided into three groups based on their solubility: sarcoplasmic (e.g., myoglobin), myofibrillar (e.g., myosin and actin) and stromal proteins (e.g., collagen and elastin) [1]. Some proteins derived from muscle and other animal tissues are used as functional (technological) ingredients in food processing (e.g., collagen  and gelatin, and beef plasma protein). In addition to traditional sources of meat, there are many prepared meat products that include muscle proteins from a variety of sources. The best known product of this group is surimi, which is a crude myofibrillar protein concentrate prepared by washing minced, mechanically deboned fish muscle from under-utilized marine fish, or mechanically separated chicken meat or animal by-products (e.g., beef heart muscle) [1]. 

*Milk proteins* make up 3.5% of cow milk and also comprise a heterogeneous group of proteins, which are represented by two major groups: caseins (80%) and whey proteins (20%). Caseins are phosphoproteins and exist in milk as large colloidal aggregates, comprised by α_s1_-, α_s2_-, β- and κ-caseins and known as casein micelles, while whey proteins, represented by β-lactglobulin, α-lactalbumin, serum albumin, immunoglobulins, lactoferrin and proteose-peptone fractions, are molecularly dispersed in the solution. Among milk proteins, only κ-casein contains about 5% of carbohydrates (tri- or tetrasaccharides), consisting of *N*-acetylneuraminic acid (sialic acid), galactose and *N*-acetylgalactosamine [2]. During cheese-making, κ-casein is hydrolysed by chymosin into para-κ-casein (residues 1-105), which remains with the curd, and caseinomacropeptide (residues 106-169), which becomes a part of sweet whey. 

Milk proteins are ingested not only with dairy products, but also as liquid, concentrated or dried food supplements or ingredients widely available on the market [3]. Heterogeneity and complexity of milk proteins, determined by their distinct biological and physicochemical properties, provide a wide spectrum in their application as bioactive functional ingredients to regulate food intake and metabolism [4,5,6].

*Egg proteins* comprise about 13% of whole egg content, and are morphologically divided into proteins of egg white (albumen) and yolk. Ovalbumin, ovotransferrin (conalbumin) and ovomucoid are the most abundant proteins of egg white where their content is 54, 12-13 and 11%, respectively, while the rest 12-13% are minor proteins (e.g., lysozyme, G2- and G3-globulins, ovoinhibitor, cystatin, avidin and others). Egg yolk, when separated by centrifugation, comprises proteins of sedimented granules, and supernatant (plasma). Proteins of yolk granules include major fractions: α- and β-lipoproteins (70%), phosvitin (16%) and low-density lipoprotein (LDL) (12%), and minor proteins such as lipovitellin, phosvitin and vitellogenin, while yolk plasma contains LDL, livetins, yolk riboflavin-binding protein and biotin-binding protein [7]. Although egg white is an excellent source of high quality protein, it contains ovoinhibitor, the serine proteinase inhibitor that can inhibit digestive enzymes such as trypsin and chymotrypsin. This might be an important factor influencing the regulatory functions of the GI tract, especially when raw egg whites are consumed or used for some food applications without thermal processing.

*Plant proteins* are also complex and cereal, pulse and legume proteins differ in characteristics.

*Cereal proteins* from wheat, rye, triticale, barley, maize, sorghum, rice, oat and millet are composed of heterogeneous groups of proteins. The amount of protein varies among cereals from as low as 8% in rice to 12% in wheat. Cereal proteins, based on their biological functions, are divided into two classes: metabolically active (cytoplasmic) proteins and storage proteins. The metabolically active proteins of cereals encompass mostly enzymes including protease inhibitors, while storage proteins are divided into albumins and the globulins, prolamins and glutelins. The storage proteins contain a large proportion of glutamic acid and proline, and only a small proportion of lysine, arginine and threonine and tryptophan. In contrast, the metabolically active proteins contain less glutamic acid and proline, and more lysine and arginine. Therefore, they have a higher nutritive value than storage proteins that mostly represent endosperm protein, which is the main protein flour [8]. Even though the amino acid content varies among species of grains, lysine is the first and tryptophan is the second limiting amino acid among all grains.

*Pulse proteins* originate from edible seeds of legumes (plants with a pod), which include dry peas, beans, lentils and chickpeas. Pulses contain 17-30% of protein, and the major proteins found in pulses are globulins (legumin and vicilin) and albumins (enzymatic proteins, protease inhibitors, amylase inhibitors and lectins). Unprocessed pulse seeds contain anti-nutritional factors (e.g., trypsin and chymotrypsin inhibitors), which decrease protein digestibility if not properly inactivated during processing [9]. 

*Soy proteins* are derived from soy beans, which have high protein content (35-40% of dry weight) [10]. Approximately, 90% of the proteins in soybeans exist as storage proteins, primarily β-conglycinin, a glycoprotein composed of three sub-units, and glycinin, a hexameric protein, where each sub-unit is composed by acidic and basic polypeptides linked together by a disulfide bond [11]. 

*Proteins from oil-producing plants other than soy*: Storage proteins represent the biggest protein fraction in oilseed crops, including cruciferin or 12S protein in canola or rapeseed, zein in corn, 11S protein in cottonseed, 12S protein in flax and hemp, arachin in peanut, carmin in safflower, α-globulin in sesame, helianthin in sunflower and others [12]. There are many proteins isolated from oil-producing crops and proved to be suitable for human consumption. All such crops contain a mixture of various proteins (albumins, globulins and glutelins) with different biological functions.

### 2.2. Composition

Many proteins are combined with non-protein components (conjugated proteins), and this affects their physiological functions. Casein, as major protein in bovine milk, contains substantial amount of phosphorous and calcium in micellar form. Many bioactive components including flavonoids, lectins, saponins, phytates, cysteine proteases and trypsin inhibitors contribute to the physiological functions of plant proteins. Soy proteins are closely associated with minor components such as isoflavones, saponins, trypsin inhibitors, phytic acid, lectin, fibres, and others. Isoflavones (e.g., genistin, daidzin, and glycitein) are major soy phytoestrogens present in soy foods [11]. While trypsin inhibitors impact pancreatic function and growth in animal models, isoflavones influence the reproductive system [13], glucose metabolism [14,15], cancer [16] and cardio-vascular diseases [17]. 

### 2.3. Processing and Matrix Effects

Due to their polarity, most proteins are soluble in water and alcohol. However processing influences the bioavailability and physico-chemical properties of proteins, including solubility, heat lability and isoelectric pH, and thereby influences their functional and physiological properties [18,19]. Various processing techniques have been applied to facilitate the integration of proteins in food products and to improve their nutritional values. Texturization, glycosylation, chemical modification including acylation and alkylation, and enzymatic modification including dephosphorylation and plastein reactions (e.g., adding or eliminating selected amino acids by formation of peptide bonds) are the most common processing techniques [20]. Texturization is a method that confers a fibre-like structure to globular proteins and has been applied to many proteins with globular structure (e.g., soy protein, wheat gluten, and oilseed meals) [21]. Glycosylation also constitutes an important processing technique to improve the functionality of proteins in food matrix. Glycosylation of vicilin in pea protein increases its solubility, emulsifying capacity and emulsion stability [22]. Glycosylation may also influence the physiological properties of proteins. For example, many physiological roles of caseinomacropeptide (CMP), a sub-fragment of κ-casein, are primarily attributed to its glycosylated form, glycomacropeptide (GMP) [23].

Processing can also be a determinant of the amount and the composition of the non-protein components: For example, defatted soy flour, which is produced by grinding the defatted flakes, contains 50% protein, while soy protein concentrate, traditionally made by aqueous alcohol extraction of defatted soy flakes, contains 70% protein, and soy protein isolate, produced by alkaline extraction of the flour followed by precipitation at acid pH, contains more than 90% protein. The concentration of isoflavones in toasted soy flour is the highest (~3095 μg/g), followed by isolated soy protein (2161 μg/g) and textured soy protein (~2109 μg/g) [24].

Although various processing methods (e.g., thermal treatment, high hydrostatic pressure and fermentation) elicit favorable effects on dietary proteins by facilitating their integration into various food products and by increasing their digestibility and peptide release, negative effects of thermal heating and dehydration on protein hydrolysates and bioactive peptides (BAPs) have also been reported [25].

These chemical and other structural characteristics of proteins as they exist in natural form, in processed foods, or in purified forms have many physiological effects beyond the provision of amino acids for protein synthesis. The primary impact of these characteristics occurs in the GI tract. 

## 3. Fate of Dietary Proteins in the Gastrointestinal Tract

Dietary proteins influence mechanical, hormonal and neuroendocrine functions of the GI tract throughout their digestion, absorption and post-absorption processes. The interaction of protein-digested products with enteric nerves and endocrine systems in the GI tract influences their digestion and absorption kinetics and ultimately the metabolic and physiological fate of proteins. 

### 3.1. Digestion and Absorption of Proteins

#### 3.1.1. Digestion

Dietary proteins and peptides are subjected to complex changes during ingestion, digestion and absorption. First, hydrolysis of proteins by proteinases (e.g., gastric pepsin and pancreatic trypsin and chemotrypsin) results in a pool of peptides, some of which are biologically active. The peptides may be further hydrolyzed by peptidases in the pancreatic secretions to small peptides that are digested by brush-border peptidases at the surface of the epithelial cells to produce free, di-, tri- and oligo-amino acids, or are absorbed. Proteins and peptides present at different stages of digestion may express a variety of functions in the GI tract [11,26]. These include the regulation of digestive enzymes, the modulation of nutrient absorption in the intestinal tract and other post-absorptive metabolic signals. For example, glycomacropeptide (GMP) suppresses protein hydrolysis by inhibiting gastric secretion and motility [23].

The site of digestion varies depending on the source of proteins. For example, casein, the major protein in the bovine milk, precipitates in the stomach where it is hydrolyzed, whilst whey and soy protein, which are soluble proteins, pass rapidly through the stomach and undergo digestion by pancreatic enzymes. Because gastric pepsin and pancreatic trypsin have different affinity for bonds between amino acids, the site of digestion determines the type of peptides produced by enzymatic hydrolysis and consequently their further physiological properties. Pepsin cleaves peptic bonds next to aromatic amino acids such as phenylalanine, tryptophan, and tyrosine [27], while trypsin cleaves bonds next to the basic amino acids arginine and lysine. Therefore, unique physicochemical properties (e.g., micelles) as well as amino acid sequence of each dietary protein predetermine the course of proteolysis and composition of peptides released. 

#### 3.1.2. Absorption

As with glucose transport, *in vitro* experiments have shown that active amino acid transport is sodium-dependent in a gradient-dependent manner across the brush border membrane of intestinal epithelial cells [28]. Variation in the absorption rate of individual amino acids has been shown and this rate varies among species [29]. In addition, some amino acids may be transported by more than one mechanism (e.g., glycine, proline and hydroxyl proline), making the kinetic absorption of amino acids difficult to predict. 

In addition to the rate of digestion and absorption of amino acids, utilization of amino acids by splanchnic tissues is a major factor determining the bioavailability of amino acids for other tissues and organs. The small intestine, metabolically, is one of the most active tissues in the body [30]. Utilization of amino acids by splanchnic tissues is highlighted by the fact that the whole body requirements for specific amino acids is 30% less with parenteral feeding compared with enteral feeding in both humans [31] and animals [30]. For example, several non-essential amino acids including glutamine, glutamate and aspartate are extensively oxidized in entrocytes, such that almost all in a conventional diet do not enter the portal vein [32]. The circulating glutamine is synthesized from branched chain amino acids (BCAA) and α-ketoglutarate in tissues [33]. In humans, the splanchnic tissues retain between 20-50% of the dietary intake of specific essential amino acids [34,35]. However, BCAA are primary exceptions, with approximately 80% of dietary protein content appearing directly in blood circulation [36]. In piglets fed milk protein, 40% of leucine and valine and 30% of isoleucine in the diet were found to be absorbed by the portal-drained viscera, with less than 20% of the absorbed BCAA being utilized for protein synthesis in intestinal mucosa [37]. 

### 3.2. Physiological Significance of Bioactive Peptides in the GI Tract

Many of the biological and physiological actions of dietary proteins have been attributed to the BAPs encrypted in protein molecules [38]. 

Digestion and absorption play a key role in the formation and degradation of BAPs [39]. Numerous active peptides are produced during the digestion of dietary proteins in the stomach and small bowel [40]. Some BAPs are hydrolyzed during digestion to shorter active forms, such as the conversion of the peptide beta-casein f(133-138) Leu-His-Leu-Pro-Leu-Pro (LHLPLP), a peptide identified in milk fermented with *Enterococcus faecalis*, to His-Leu-Pro-Leu-Pro (HLPLP) prior its transportation across the intestinal epithelium [41]. HLPLP has been detected in human plasma after the consumption of a fermented milk enriched with ACE inhibitory peptides [42]. Moreover, the active form of milk-derived peptide Lys-Val-Leu-Pro-Val-Pro-Glu (KVLPVPQ) is formed by hydrolysis of glutamine during pancreatic digestion [43]. 

To be biologically active, it is essential for BAPs to survive proteolysis in the luminal contents of the GI tract. The stability of several BAPs have been reported, including casomorphins [44], somatostatin [45], lactoferrin [46] and Epidermal Growth Factor (EGF) [47]. It is still not clear which exact characteristics do prevent the breakdown of these BAPs. The stability of BAPs may also vary within a class. The beta-casomorphin-4 (Tyr-Pro-Phe-Pro-NH_2_) showed no effect on GI transit time, while all other types increased GI transit time in a dose-dependent manner [44]. Thus, the authors suggested that the latter were more resistant to proteolytic attack and had a higher opioid potency than beta-casomorphin-4. 

Dietary proteins and peptides may also influence the stability of endogenous peptides and consequently influence their physiological functions. For example, protein-derived components in the digesta enhance cholecystokinin (CCK) release by preventing the degradation of duodenal CCK-releasing factor (CRF), acting as substrates for trypsin. Similarly, trypsin inhibitor compounds, which are abundant in soy protein, increase CCK release in both rats [48,49] and humans [50,51] by inhibiting the degradation of CRF by trypsin.

The interaction between protein-digested products and gut peptides, such as CCK, Glucagon-Like Peptide 1 (GLP-1), peptide YY (PYY) and ghrelin, also influences GI tract functions and determines the metabolic responses to ingested proteins. Hydrolysate forms of casein have a greater effect on food intake via CCK-A and opioid receptors than their intact form [52], suggesting a role for casomorphins, which have an opioid-like activity, and of GMP, which stimulates the release of CCK [53,54]. The progressive and sequential emptying of gastric products also plays an important role in the regulation of gastric and pancreatic secretions [55]. Yvon and colleagues reported that caseinomacropeptide (CMP) is almost the only peptide released from the stomach during the first hour after casein ingestion [23]. GMP, the glycosylated form of CMP, inhibits gastric secretion and motility [56]. The modulating role of CMP on CCK, gastrin and somatostatin release may constitute one of the potential mechanisms through which casein protein affects gastric and pancreatic secretions as well as GI motility [57,58]. GMP also suppresses protein hydrolysis and amino acid absorption by inhibiting gastric secretion and motility [23]. Similarly, β-conglycinin, a peptide derived from soy protein, stimulates CCK release [59], and delays gastric emptying and suppresses food intake [59,60]. 

Whether the effect of exogenous peptides is limited to their pre-absorptive action in the GI tract or remains after they cross the lumen and enter blood circulation is uncertain. Some studies report that the mammalian small intestine can absorb gamma-globulins and other milk proteins [61], while some others reported limited post-absorptive bioavailability of peptides [62]. In humans, the peptides Valine-Proline-Proline (VPP) and Isoleucine-Proline-Proline (IPP) were detected undegraded in the plasma following the consumption of a lactotripeptide-enriched yogurt beverage [63]. 

Several mechanisms for absorption of peptides through epithelial cells have been suggested. Proteins and oligopeptides can be transported by transcytosis (vesicle-mediated transcellular transport) [64]. However, more than 90% of the proteins and oligopeptides are hydrolyzed intracellularly [65]. Di- and tripeptides are actively transported via a specific transporter (PepT1) [66], but are also further hydrolyzed to amino acids in the epithelial cells. Proteins and peptides may also be passively transported via the paracellular route [67], which is a passive diffusion system that employs a space between intestinal epithelial cells [68]. Paracellular transport is regulated by the permeability of tight junctions, and some nutrients, including proteins (e.g., β-lactoglobulin), can act as regulators of tight junction-mediated permeability through cell signaling mechanisms. Such mechanisms involve the recognition of nutrients by intestinal epithelial cell receptors, followed by signal transduction to tyrosine kinase, phospholipase C and protein kinase C pathways and resulting in cytosceletal changes in tight junctions [69]. This suggests that protein digesta may act synergistically with some proteins regulating the absorption rate, while other peptides have post-absorptive effects. Under certain pathological conditions, paracellular permeability is increased and this, in turn, can increase mucosal immune activity, enhance disease progression and severity and, possibly, be a risk factor for development of diseases [70]. This also suggests that the absorption rate of intact proteins and peptides may depend on the maturity and health status of the individual.

Progress in identifying mechanisms of peptide absorption was made by the utilization of Caco-2 human intestinal epithelial cells, which form a monolayer with tight junctions between cells, microvilli, receptors, and expressed enzymatic and transporter systems attributable to those cells *in vivo*. Studies with Caco-2 cell lines found that only 2% of the intact tripeptide, VPP, was absorbed and detected on the basal side of the monolayer by a paracellular route, while the rest was digested either by brush border or intracellular peptidases, including that transported via the peptide transporter, PepT1, and hydrolyzed by intracellular peptidases [71]. Supporting evidence for limited transport of intact peptides has also been provided by an *in vivo* study in conscious pigs. The presence of intact proline-rich tripeptides VPP and IPP in the blood circulation was estimated to be approximately 0.1% of that administered through intragastric infusion via a saline solution.

Nevertheless, VPP and IPP are naturally present in many fermented dairy products and their consumption has been shown to lower blood pressure and inhibit angiotensin-converting enzyme (ACE), the component of rennin-angiotensin system (RAS) [72]. Half-lives of absorption and elimination for these peptides were about 5 and 15 min respectively [62]. Absorption of intact proteins and peptides was also shown in a study with dogs exposed to intraduodenal infusion of somatostatin. Increased concentrations of somatostatin were found in blood [73]. Evidence for absorption has also been shown by physiological measures. Oral administration of a daily dose of casein hydrolysate containing tripeptides VPP and IPP (0.49 g/day) in humans and in hypertensive rats (32 mg/kg BW/day) lowered blood pressure [64,74]. Therefore, the physiological effects of BAPs may be due to one of efficacy at a very low concentration, an accumulation of an active concentration in certain tissues (e.g., aorta) [75,76,77], an interaction with gut wall or luminal receptors without being absorbed, or a combination of these actions.

## 4. Protein Sensing in the GI Tract

The digestion and absorption of proteins in the GI tract affect their physiological and metabolic properties and occur through a complex monitoring and control system. The GI tract is the largest neuroendocrine organ in the body, with enteric nervous and entero-endocrine systems that are integrated within the GI tract and extend to specific areas of the brain [78]. 

The GI tract endocrine system produces a large number of peptidergic hormones and regulatory factors, localized mainly in the mucosa of the GI tract from the stomach to the rectum and in the pancreas. Dozens of hormones and paracrine factors are secreted from the GI tract in response to a meal [79], and some of the endocrine cells are directly exposed to ingested bioactive compounds in the lumen [80]. Many of the activities of the GI tract, including GI motility, gastric emptying and gastric and pancreatic secretions, are under the control of gut peptide hormones that are released in response to nutrients in the GI tract [81]. Many gut peptides are also found in the brain and are components of the gut-brain axis that regulates food intake and metabolic functions including blood glucose control [80]. 

The enteric nervous system (ENS) of the GI tract communicates with the central nervous system (CNS) through the parasympathetic (e.g., via the vagus nerve) and sympathetic (e.g., via the prevertebral ganglia) nervous systems [82]. Many of the gut peptides act on GI receptors that relay messages to the brain via the afferent vagus nerve [83,84]. Some of the GI hormones, such as CCK, PYY, neuropeptide Y (NPY) and somatostatin, originally believed to act on the target digestive organs via endocrine pathways, also utilize neural pathways to exert their biological actions [80]. 

Signaling of protein ingestion begins in the oral cavity and finishes in the colon. Therefore, pregastric, gastric, and post-gastric signals are important in determining the physiologic outcomes of protein ingestion.

### 4.1. Oral and Gastrointestinal Chemosensing

The chemosensing of the alimentary tract serves to recognize whether the response to stimulus (nutrient or toxin) should be defensive (e.g., vomiting) or nutritional (digestion, absorption and storage) [85]. Protein sensing begins with the taste receptors. 

The tongue and the upper GI tract sharecommon systems for nutrient sensing that are mediated through the vagus nerve, which is extensively distributed throughout the GI tract, from the posterior region of the oral cavity and esophagus to the lowest part of the colon. It functions as the primary neuroanatomical circuit in the gut-brain axis to transmit meal-related signals from the GI mucosa to the central nervous system. Recent findings indicate that taste receptors (T1R and T2R) of taste bulbs are also expressed in the GI mucosa. Conversely, receptors for well-knowngut neurotransmitters (*i.e.*, serotonin (5-HT)) and hormones (*i.e.*, CCKand Vasoactive Intestinal Peptide (VIP)) are also expressed in taste buds of the oral cavity. Thus, regulatory processes (e.g., ingestive behavior, nutrient absorption, GI secretion, and stomach emptying) as well as conscious sensations (e.g., satiety, nausea, and discomfort) begin immediately with food contact [86].

Some food proteins and many hydrolysates have a bitter taste due to the presence of peptides whose terminal amino acids are hydrophobic, such as isoleucine, tyrosine, phenylalanine, and tryptophan. The bitter taste is related to their hydrophobicity, while in intact proteins, hydrophobic amino acid residues usually remain inside the molecule and do not interact with taste receptors when ingested with food. However, bitterness also decreases at high degrees of conversion, suggesting that bitterness can be decreased by cleaving bitter peptides into free amino acids [87].

Humans perceive different amino acids in the oral cavity as umami (savory), sweet, bitter, and sour. Amino acids infused into different parts of the GI tract, including stomach, duodenum and portal vein, may either activate or inhibit vagal afferent activities [85]. For example, intraportal administration of Alanine, Arginine, Histidine, Leucine, Lysine, Serine, Tryptophane, and Valine, increased, while Cysteine, Glycine , Phenylalanine, Proline and Threonine decreased vagal afferent discharge rate [88]. However, when 20 different amino acids were infused into the stomach, only glutamate affected gastric vagal afferent activity. The effect of glutamate was suggested to be mediated through serotonin receptors (5-HT_3_) [86]. Similarly, when infused into the duodenum, glutamate was found to increase vagal afferent activity [89]. 

Chemical sensing between dietary proteins and enteroendocrine cells is partially mediated by the G protein-coupled family of receptors (T1R and T2R). Such interactions between nutrients and sensitive cells trigger a complex response that involves the release of GI peptides, with further activation of visceral vagal afferent neurons through the paracrine action. T1Rs receptors mediate either sweet taste or sense amino acids or umami, while T2Rs mediate bitter taste [90]. These receptors have been found in the taste buds of the lingual epithelium and in different areas of the GI tract including gut epithelium and pancreas. 

The T1R system has broad specificity for sweet-sensing and can be activated by natural sugars, sweet proteins and artificial sweeteners. In response to glucose, T1Rs stimulate the release of GLP-1 and Gastric Inhibitory Polypeptide (GIP), and upregulate the sodium-dependent glucose co-transporter (SGLT-1) in the intestinal epithelium [91,92]. Secreted GLP-1 interacts with GLP-1 receptors expressed on vagal afferents and thereby regulates glucose homeostasis [93] and satiety [94]. 

The T2R system is believed to have evolved as a protective mechanism against food toxins by detecting bitter taste, stimulating food aversion and decreasing gastric emptying [95]. Application of ligands for the T2Rs induces rapid Ca^2+^ signaling and release of CCK [96]. Intragastric administration of either denatonium or phenylthiocarbamide (both bitter compounds) activates neurons in the nucleus tractus solitarius (NTS), the region where vagal afferents terminate, and this response is blocked by subdiaphragmatic vagotomy. Activation of the vagal pathways by denatonium is dependent on CCK1Rs and Y2Rs, receptors for CCK and PYY respectively [97,98]. Thus, activation of T2Rs have been suggested as a mechanism to decrease gastric emptying and reduce food intake [90]. However, the role of individual food proteins and their hydrolysates in the physiological effects modulated through T1R and T2R receptors, localized throughout the GI tract, has not been yet reported.

### 4.2. Stomach and Small Intestine

The stomach determines the rate of appearance and the composition of digesta in the duodenum [55]. Gastric emptying is regulated by chemical and physical characteristics of ingested meals [99], including osmolality, pH, temperature, viscosity, energy density and volume. 

Ingested proteins and the stomach influence each other in a bi-directional way. Gastric digestion and gastric emptying rates are major determinant of the bioavailability of amino acids and their subsequent utilization [100]. The relatively high importance of ingested protein as a determinant of gastric emptying rate is highlighted by the observation that formulas containing the same whey protein had similar emptying rates despite having different osmolality and caloric density, which are factors known to affect stomach emptying [100]. However, the effects of proteins differ among sources. The emptying rate of the stomach after casein meal is slower as compared with that following soy meal [101], and it is also longer after the ingestion of casein preload compared with whey preload (48 g protein) [102]. Gastric emptying rate as well as GI transit time were also found to be significantly longer after casein suspension as compared to whey suspension (0.5 mL), when given to rats by gastric tube [44]. 

The mechanism by which dietary proteins affect stomach emptying is through their effects on the release of gut hormones. CCK, PYY and GLP-1, gut peptides released in response to ingested proteins, delay gastric emptying by regulation of pyloric pressure and gastric motility [103,104,105,106]. Furthermore, GLP-1 causes a moderate and stable flow of gastric emptying via the “ileal break” mechanism [107]. In contrast, ghrelin is a gastric peptide stimulating gut motility [108]. Dietary proteins suppress ghrelin more than fat and carbohydrate [109]. While most of the described endocrine activities of GI hormones are based on the determination of their concentrations in bloodstream, blood concentrations do not accurately reflect the whole spectrum of their activities. Exocrine activities of CCK can occur through paracrine mechanisms as a result of activation of proximate vagal afferent nerve terminals [110]. Similarly, PYY may function as endocrine, paracrine, and neurocrine transmitters [111]. However, it is still not clear whether other GI hormones function through neurocrine mechanisms. 

Proteins or BAPs from them may also stimulate receptors directly. Opioid receptors, which are wide spread from stomach to distal colon in rats [112] and from jejunum to distal colon in humans [113], are a target for exogenous opioid peptides. Opioid peptides, derived from milk proteins [114], are involved in the regulation of appetite [52], plasma insulin [115] and blood pressure [116]. In addition, casomorphins exhibit an antidiarrhoeal effect by prolonging GI transit time and by enhancing net water and electrolyte absorption [117]. Furthermore, the intermediary role of peripheral opioid receptors in protein-induced satiety has been reported [52,118]. 

### 4.3. Large Intestine

The large intestine is a primary site of microbial colonization in humans due to its neutral pH, slow intestinal motility and very low oxidation/reduction potentials [119]. Ingested proteins and microbiota also affect each other in a bi-directional manner.

Microbiota in the GI tract plays a crucial role in optimum function of the GI tract and prevention of infection [120]. In addition, biosynthesis of vitamins (e.g., vitamin B12 and vitamin K) and short-chain fatty acids (principally acetate, propionate and butyrate) by microbiota is well-established [121,122]. Microbiota can also produce bioactive components from ingested foods [123]. Moreover, microbiota may influence energy homeostasis by affecting energy utilization from diet components and by altering host genes that regulate energy expenditure and storage [123]. 

Protein-derived components, and particularly peptides, have an impact on the composition of the GI tract microbiota [124]. Both source and composition of ingested proteins influence the composition and metabolic activity of the intestinal microbiota [125]. For example, it has been suggested that whey proteins, which leave the stomach quickly, are not hydrolyzed by pancreatic proteinases and could play a role in the establishment of the microbiota of the young [126]. Lactoferrin, an iron glycoprotein derived from whey protein, as well as its sub-fragment lactoferricin are known to exert a broad-spectrum primary defence against a wide range of gram negative and positive bacteria, fungi (e.g., Candida) and protozoa (e.g., *Toxoplasma gondii*) both in *in vitro* and *in vivo* models [127,128]. Anti-viral effects of lactoferricin against several enveloped and naked viruses have also been shown in an*in vivo* model [127]. Similarly, antimicrobial amino acid sequences in casein proteins have been identified. Casocidin-I derived from *αs*_2_-casein fragment, with a high proportion (10 of 39) of basic amino acid residues, have shown inhibitory effects on the growth of *Escherichia coli* and *Staphylococcus carnosus* [129]*.* Similarly, isracidin, derived from *αs*_1_-casein, was found protective against *Staphylococcus aureus* and *Candida albicans* in mice[130]. The mechanisms by which proteins and their peptides exhibit anti-microbial effects are not clear but three actions have been identified. First, it has been suggested that the net positive charge of peptides released during digestion plays a crucial role, destroying sensitive microorganisms by increasing cell membrane permeability [127]. Second, the binding of enterotoxins and the inhibition of viral and bacterial binding to tissues is another mechanism. For example, GMP has been shown to inhibit the binding of cariogenic bacteria to oral surfaces [54]. Finally, nutrient sequestering may be a factor. The bacteriostatic effects of lactoferrin are mainly due to its iron sequestering property [127]. 

Although excretion of protein in feces has been considered an indicator of low bioavailability of dietary proteins, this may underestimate the benefits of protein utilization by microbiota in the large intestine [50]. Biosynthesis of indispensible amino acids in the GI tract by microbiota may influence total nitrogen balance in humans [131]. Absorption of microbial amino acids has been reported both in pigs [132] and humans [131]. Lysine has been utilized as an indicator of absorption of microbial amino acids [133]. Between 1-20% of circulating plasma lysine, urinary lysine and body protein lysine of the host is derived from intestinal microbial sources [125].

The fermentation of proteins by microbiota may produce compounds with adverse metabolic effects or reduce their presence. The formation of toxic compounds with metabolic effects includes ammonia, dehydrogen sulphide, indoles and phenols [134]. However, luminal microbes can also deaminate amino acids, hydrolyze luminal urea and recycle this ammonia [135].

The mechanisms underlying the physiological and metabolic consequences of the interaction of ingested proteins and microbiota remain to be described. However, endogenous distal gut peptides released from the lower small intestine, such as GLP-1 and PYY, may play a role. In rodents, the fermentation of resistant starch by microbiota in the colon has been suggested as a primary mechanism for the endogenous production of distal gut peptides GLP-1 and PYY [136,137,138,139]. In humans, the consumption of prebiotics increases plasma concentrations of GLP-1 and PYY and these responses are correlated with increased breath hydrogen excretion, a marker for gut microbiota fermentation [140]. Probiotic consumption has also been shown to decrease food intake, body weight gain and fat mass development in obese subjects, consistent with the increased PYY and decreased ghrelin postprandial responses in these subjects [141]. Because GLP-1 and PYY are also known as satiety signals in response to dietary proteins [104,142], the interaction of protein-digested products with microbiota in the GI tract may constitute one of the possible mechanisms underlying the satiating properties of dietary proteins. Moreover, an interaction between microbiota and opioid receptors has been reported. Lactobacillus acidophilus was found to stimulate opioid and cannabinoid receptors [143]. 

## 5. Role of the GI Tract in Regulation of Metabolism and Food Intake in Response to Dietary Proteins

To illustrate the metabolic and physiologic consequences of the interactions between dietary proteins and the GI tract, the effects of protein ingestion on the regulation of the metabolism of amino acids, glucose and lipids and of food intake are discussed in the following.

### 5.1. Metabolism

#### 5.1.1. Amino Acid Metabolism

The source of dietary proteins affects amino acid kinetics due to several factors including amino acid composition and their effect on gastric emptying [102,144], the composition of intestinal effluents [36], rate of digestion and amino acid absorption, and oxidation [38,61,62]. 

Generally, the amount of protein synthesis after protein consumption depends on the match between the composition of indispensible amino acids in the dietary protein and the requirement for protein synthesis [145], and is offered as an explanation for the lower protein synthesis reported after the ingestion of soy protein compared with animal proteins [145]. In addition, the rate of splanchnic retention of dietary nitrogen is reported to be directly proportional to protein synthesis, explaining the greater protein synthesis observed after ingestion of fast compared with slow proteins [146].

Amino acid kinetics, arising from interaction between dietary proteins and the GI tract, has also been proposed as a reason for variations in their postprandial metabolic effects [147,148]. For example, caseins [147] delay amino acid delivery to the gut because their phosphate-containing proteins precipitate under the acidic gastric pH [144]. On the other hand [147], whey remains soluble and rapidly passes through the stomach leading to a faster delivery of amino acids to circulation. Thus, whey protein ingestion mediates larger increases in post-meal aminoacidemia than casein and leads to a higher protein synthesis, whereas casein has a greater effect on reducing protein breakdown [147]. Similarly, differences in amino acid kinetics have been reported in studies comparing milk proteins (primarily casein) to that of soy protein, a rapidly digested protein [149]. 

Although differences in the rate of gastric emptying have been suggested to explain the discrepancy in amino acid kinetics observed following the ingestion of casein and whey proteins [144], not all studies support this view. Some report a significantly slower rate of gastric emptying after casein ingestion as compared to whey protein [102,144], while others described no difference [99]. An explanation for this inconsistency may reside in the methods used to measuring gastric emptying rate [99,144]. Slower gastric emptying of casein was found when flow rate of the liquid effluents and the nitrogen movements after milk protein ingestion was measured by perfusion of saline solution containing phenol red [144], while no differences were found in gastric emptying rate when determined by measuring plasma concentrations of paracetamol added to liquid preloads [99].

Postprandial amino acid profiles, reflecting the amino acid composition of dietary proteins in addition to their rates of digestion, absorption and first pass metabolism in the enterocytes and liver [102], are also influence the metabolic and physiological consequences of ingested proteins [99,147,149]. A greater increase in blood essential amino acids concentrations following the ingestion of whey protein in comparison to either casein or soy protein [150] has been offered as the explanation for the higher rate of protein synthesis reported in young men after whey proteins. In addition, the higher concentrations of indispensable amino acids following the consumption of whey protein as compared with casein may lead to higher rates of amino acid deamination and therefore less protection against protein breakdown [146,151]. However, individual amino acids have been proposed as factors altering protein synthesis. For example, BCAAs and particularly leucine, which represents around 12% of the amino acids in whey protein, independently stimulate muscle protein synthesis [152]. Whey also contains higher concentrations of total sulfur amino acids (methionine and cysteine), lysine, threonine, and tryptophan compared to either casein or the complete mix of proteins in milk [146], but their individual contribution to the effect of whey protein on protein synthesis has not been explored.

#### 5.1.2. Glucose Metabolism

The interactive effect of dietary proteins and glucose metabolism is well established. The effect of proteins on glucose metabolism depends on amino acid composition, digestion kinetics of proteins and amino acid utilization in the GI tract. When consumed with carbohydrates, dietary proteins reduce glycemic responses [153,154]. Amino acids released from proteins have both direct (substrate-mediated) and indirect (hormone-mediated) effects on glucose metabolism [155]. Postprandial elevation of plasma amino acids increase the secretion of both insulin and glucagon [156,157] and, therefore, alter hepatic glucose metabolism by changing the portal insulin/glucagon ratio [158]. 

Arginine and the BCAA leucine are known as the most potent amino acids in stimulating pancreatic insulin release and therefore decreasing plasma glucose concentration. Leucine also stimulates intracellular insulin signaling in muscles and adipose tissue [159]. Similar effects of ingestion of whey, whey hydrolysate or BCAA alone on plasma insulin and glucose concentrations suggest that the effect of ingesting whey protein is due to its BCAA content [160]. In addition, the ketogenic and glucogenic amino acid content of proteins reflect their effect on gluconeogenesis and consequently glucose utilization [145].

The rate of protein digestion is also a factor impacting their effect on glucose metabolism. Ingestion of proteinase inhibitors delays gastric emptying and decreases glucose and insulin plasma concentrations in type II diabetic patients [161]. Therefore, the rapid digestion of whey and the resulting increase in insulin release explain in part its greater effect on increasing glucose uptake by tissues and reducing plasma glucose concentrations compared with the ingestion of other proteins, including casein, that are more slowly digested [99,147].

#### 5.1.3. Lipid Metabolism

Ingestion of dietary proteins also contributes to the regulation of lipid metabolism in both humans [162,163] and animals [164,165], but their effects are source-specific.

Most studies of dietary proteins and peptides have focused on plasma cholesterol concentrations [164,165,166,167,168]. Whey protein has a greater hypocholesterolemic effect than either casein or soy protein in rats [168]. Favourable effects of soy protein on plasma lipid profile have been reported [169], but may be in part due to its non-protein bioactive components (e.g., isoflavones) [170]. Although egg intake has been reported to increase total serum cholesterol in both animals [171] and humans [172], recent studies of egg white proteins [173] and of ovomucin, one of the egg white proteins [174], show decreased serum cholesterol concentrations in rats. Beef proteins were found to increase plasma cholesterol levels in rabbits [171], have no effect in rats [175] or decrease in rats fed a cholesterol-enriched diet [164]. In contrast, fish protein did not show consistent effects on cholesterol levels in either rats [164] or rabbits [165].

Although the mechanisms by which dietary proteins affect cholesterol metabolism are unclear, their effect may be due in part to their BAPs and amino acid composition and to their bile-acid-binding capacity and hydrophobicity. The importance of BAPs is suggested by the observation that a high-molecular-weight 11S fraction of soy protein decreased plasma cholesterol and increased fecal excretion of steroids to a greater extent than its low-molecular-weight fraction [176]. Similarly, a cholesterol-lowering effect of the major fragments of whey protein, α-lactalbumin and β-lactaglobulin and their hydrolysates, has been reported [166]. 

Protein-digested products, including amino acids, may also play a role in lipid metabolism. Soy protein hydrolysates, which were exhaustively hydrolyzed by various methods (microbial or chemical hydrolysis), lowered plasma cholesterol more than intact soy protein [176]. High levels of ketogenic amino acids in an amino acid mixture were found to influence plasma lipid profile by increasing LDL and very low density lipoprotein (VLDL) fragments [177]. Moreover, a significant negative correlation between blood cholesterol levels and cysteine content of dietary proteins has been described [178].

Bile-acid-binding capacity and hydrophobicity may partly explain the hypocholesterolemic properties of soy protein [166]. Soy protein inhibits the formation of mixed micelle lipids and bile salts, which can subsequently suppress the intestinal absorption of cholesterol and bile acids. On the other hand, no relationship was observed between the bile-acid-binding capacity and the hydrophobicity of whey protein and its hypocholesterolemic action [166].

In summary, dietary proteins interact with the physiological functions of the gut to control amino acid, glucose and lipid metabolism. In addition, they are significant factors in determining the role of the gut in food intake regulation. 

### 5.2. Food Intake

The satiety effect of proteins is greater than fat and carbohydrate (cal/cal) [179]. The GI tract plays an important role in the interplay between exogenous proteins and peptides and endogenous peptide [142]. Protein-induced satiety has been associated with the release of gut peptides such as CCK [180], PYY [181], and GLP-1 [182]. Dietary proteins and protein-digested products mediate the release of endogenous gut peptides by stimulating chemoreceptors [183] and/or by activating satiety hormone receptors [52,142,184] and others [59,118]. Again, protein source and composition are factors for satiety signals. For example, proteins and peptides arising from the digestion of casein and soy protein suppress food intake through opioid and CCK-1 receptors [52], whereas those from whey and casein interact with exendin-4, a GLP-1 receptor agonist, to suppress food intake in rats [142]. In humans, whey protein suppresses short-term food intake to a greater extent than egg albumen and soy protein isolate in young men [185]. 

The variable effects of dietary proteins on food intake may be due to the differences in their structure, their amino acid composition and the bioactive peptides encrypted in their amino acid sequences. Protein source influences digestion, gastric emptying [186,187], and the composition of intestinal effluents [188]; the rate of amino acid absorption and oxidation [147,189,190]; plasma and brain amino acid concentrations and patterns [191,192]; and insulin and glucagon release [193,194]. All have been associated with decreased food intake. 

The effect of individual amino acids and BAPs as protein-digested products on food intake has been reported. Hydrolysates of proteins suppress food intake more than intact protein [52], suggesting a role for both free amino acids and BAPs. Elevated plasma concentrations of individual amino acids can directly influence certain areas of the hypothalamus involved in food intake regulation. The precursor roles in the brain of increased tryptophan and histidine on the synthesis of the neurotransmitters serotonine and histamine, and of tyrosine on the neurotransmitters dopamine and norepinephrine, have been shown [195,196,197]. The intracerebroventricular administration of leucine decreases food intake and body weight in rodents although the mechanism is unclear [198,199]. 

Evidence supporting activity of dietary protein derived BAPs on satiety and food intake has been reported. For example, casein-derived GMP suppressed food intake through the first half an hour of food intake more than whey, casein and complete milk protein in rats [200]. In humans, whey protein containing GMP decreased energy intake to a greater extent at three hours following breakfast consumption than whey protein alone [201]. The satiety effect of β-conglycinin, a peptide originated from soy protein, has been also shown in rats [60,202].

It is clear that the interaction of dietary proteins with regulatory functions of the GI tract have profound effects on physiologic and metabolic responses. However, at the present time, there is no standard method for describing physiologic properties of dietary proteins, analogous to the glycemic index used to describe the metabolic characteristics of carbohydrates.

## 6. Assessment of Protein Quality

Historically, many methods have been used to rank protein quality based on their digestibility, absorption of amino acids and capacity to provide metabolically active nitrogen and amino acids to tissues and organs. Such methods integrate the estimates of experimentally-derived amino acid requirement with the knowledge of the capacity of food proteins to efficiently meet these amino acid requirements. Amino acid requirement studies have traditionally used plasma amino acid concentrations, amino acid oxidation and amino acid balance methodologies [203]. Recently, the indicator amino acid oxidation (IAAO) method, using 14C- or 13C-carbon labelled amino acid oxidation, has been applied to determine almost all indispensable amino acid requirements in adult humans [204]. It is based on the concept that excess amino acids cannot be stored and therefore must be partitioned between incorporation into proteins or oxidation. When one of the indispensable amino acids is deficient for protein synthesis, all other amino acids, including the indicator labelled amino acid, are available in excess and are consequently oxidized. Thus, with increasing intake of the limiting amino acid, oxidation of the indicator amino acid will decrease, reflecting an increased incorporation into protein synthesis. The requirement for the amino acid under test is determined by the amount after which no further increases bring about a reduction in the oxidation of the indicator amino acid.

Protein quality assessment methods measure the effects of protein ingestion under controlled conditions on nitrogen excretion, growth, and protein synthesis or calculate protein quality based on amino acid composition of the test protein compared to a reference standard. The Protein Digestibility-Corrected Amino Acid Score (PDCAAS) is the currently recommended method of choice for routine assessment of protein quality of foods to meet human requirements [205]. It is based on the combination of an age-related amino acid reference pattern, which is reflective of estimates of human requirements, and of protein digestibility. It has been noted previously that the estimates of protein quality fail to consider the physiological activities of protein-derived components as determinants of amino acid utilization for protein synthesis [206]. 

Perhaps, equally worth noting is that the accuracy of amino acid requirements based on the IAAO method may be questioned for this reason. It also fails in its methodology to mimic the multitude of effects of protein ingestion on protein synthesis through physiologic effects independent of their role of providing amino acids for protein synthesis. Determination of amino acid requirements through the IAAO method is based on feeding defined amino acid mixtures to test subjects over eight hours [204]. Thus, estimates of amino acid requirements may not reflect the true amino acid requirement as influenced by physiological and metabolic effects of protein ingestion before amino acid absorption occurs. To our knowledge, there are no reports comparing the effects of acute or chronic consumption of an intact protein with an amino acid mixture of the same amino acid composition on gut hormone and metabolic responses and protein synthesis and degradation.

## 7. Summary and Conclusion

The importance of the interaction of the chemical and physical structure of food proteins with the regulation of metabolism and food intake has received considerable attention in the past fifteen years. This review has illustrated the importance of the interaction of dietary proteins during digestion and absorption with the physiological and metabolic functions of the GI tract, in the regulation of amino acid, glucose, lipid metabolism, and food intake. Dietary proteins and their digested products interact with the regulatory functions of the gastrointestinal (GI) tract in a source dependent manner. Within the GI tract, dietary proteins and their products of digestion affect several regulatory functions by interacting with receptors releasing hormones, affecting stomach emptying and GI transport and absorption, transmitting neural signals to the brain, and modifying the microflora. The characteristics of proteins including their physico-chemical properties, amino acid composition and sequence, bioactive peptides, digestion kinetics and also non-protein bioactive components conjugated with them influence their interaction with the GI tract. Yet, there are currently no assessment methods designed to evaluate the physiological quality of proteins beyond providing indispensable amino acids and contributing to protein synthesis. Clearly, the role of dietary proteins as determinants of health has to be understood beyond that provided by the traditional methods of assessment of protein quality.
